# Givosiran for the Treatment of Hereditary Coproporphyria in Pregnancy: A Case Report

**DOI:** 10.7759/cureus.115402

**Published:** 2026-08-29

**Authors:** Tess Van Schoor, Shea A Nagle, Randall Y Chan

**Affiliations:** 1 Department of Obstetrics and Gynecology, The University of Arizona College of Medicine, Tucson, USA; 2 Department of Pediatrics, Boston Children's Hospital, Boston, USA; 3 Department of Pediatrics, University of Southern California Keck School of Medicine, Los Angeles, USA

**Keywords:** givosiran, hereditary coproporphyria, porphyria, pregnancy, shared decision making

## Abstract

Hereditary coproporphyria (HCP) is a rare autosomal dominant disorder of heme synthesis characterized by acute neurovisceral attacks that are often precipitated by exposure to medications or hormones. Patients with HCP who become pregnant have an elevated risk of more frequent neurovisceral attacks and other obstetric complications. Givosiran, a small interfering RNA therapeutic, is considered the gold-standard prophylactic treatment for acute hepatic porphyrias, but its use has not been studied during pregnancy. We describe our patient who successfully continued givosiran throughout pregnancy. This highlights the role of shared decision-making in managing medically complex patients during pregnancy when treatment data may be limited or non-existent and the need for further investigation into the safety profile of givosiran use in pregnancy.

## Introduction

Hereditary coproporphyria (HCP) is a rare autosomal dominant disorder of heme synthesis characterized by acute neurovisceral attacks [1]. This mutation in the *CPOX* gene results in toxic porphyrin accumulation when hepatic δ-aminolevulinic acid synthase (ALAS) expression is induced by hormones or medications [2]. Individuals affected by HCP exhibit a significant increase in urinary porphobilinogen (PBG) during an acute attack [3].

Givosiran, the current gold standard in treatment for HCP, is a small interfering RNA (siRNA) therapeutic targeting translation of the hepatic aminolevulinate synthase 1 gene (*ALAS1*) that decreases the production of neurotoxic heme precursors by disrupting heme synthesis through the reduction of hepatic ALAS levels [4]. Givosiran is associated with a statistically significant decrease in attack frequency as well as yearly hemin use. In contrast to previous systemic treatments for porphyrias, givosiran is selectively delivered to the liver [5].

Givosiran had not been previously studied in pregnant women and is typically discontinued during pregnancy [6]. We present a patient who chose to continue givosiran after discussion and via shared decision-making, a patient-centered approach to medical decisions that facilitates collaborative treatment choices between patients and providers.

## Case presentation

The patient, nonbinary, assigned female at birth, and 19 years old at the time of pregnancy, was originally diagnosed with HCP at age 15 after many years of symptomatic flares and continued to experience monthly painful neurovisceral attacks. They were frequently hospitalized for pain control and intravenous hemin infusions. Prior to givosiran therapy, the patient had been trialed on oral and implanted hormone therapy, induced menopause using intranasal nafarelin, and chronic outpatient hemin infusions as per then-available published guidelines; all were either ineffective or poorly tolerated. Ultimately, givosiran was used to prevent porphyria attacks successfully; the patient averaged 6-12 hospitalizations per year for neurovisceral attacks prior to givosiran and has only had a single admission since initiation 4 years ago. The patient received contraception counselling and had a copper intrauterine device (IUD) placed.

Approximately eight months after IUD placement and four weeks after initiating givosiran, the patient presented to the gynecology clinic with pelvic cramping and a missed period; a urine pregnancy test was positive. Transabdominal pelvic ultrasound confirmed an intrauterine pregnancy with an estimated gestational age of seven weeks and two days. The copper IUD was noted to be partially expelled and therefore ineffective. The IUD was promptly removed, and the patient was counseled on their pregnancy options. They discussed their desire to parent a child and understood that they could potentially experience an increase in neurovisceral attacks with hormonal fluctuations during pregnancy. Available data on pregnancy outcomes for HCP patients were discussed, including increased risk of low birth weight, small for gestational age, and perinatal death.

Initially, the patient intended therapeutic pregnancy termination, so givosiran was continued; eventually, they elected to continue the pregnancy, so discontinuation of givosiran was discussed at 15 weeks of gestation. They were advised that givosiran had not been previously studied in pregnant populations and is typically discontinued during pregnancy. After discussing the unknown risks of givosiran *in utero* and the known risks of frequent porphyria attacks as well as the risks of repeated fetal exposure to high-dose opiates, the patient elected to continue givosiran therapy.

Throughout the pregnancy, the patient remained free from neurovisceral attacks. A fetal anatomy ultrasound in the second trimester was reassuring, though a small echogenic focus was seen in the left ventricle in a four-chamber view of the heart (Figure 1, echogenic focus indicated by red arrow). Maternal-fetal medicine reviewed the scan with the patient and recommended a repeat ultrasound; however, they did not obtain one prior to delivery.

**Figure 1:**
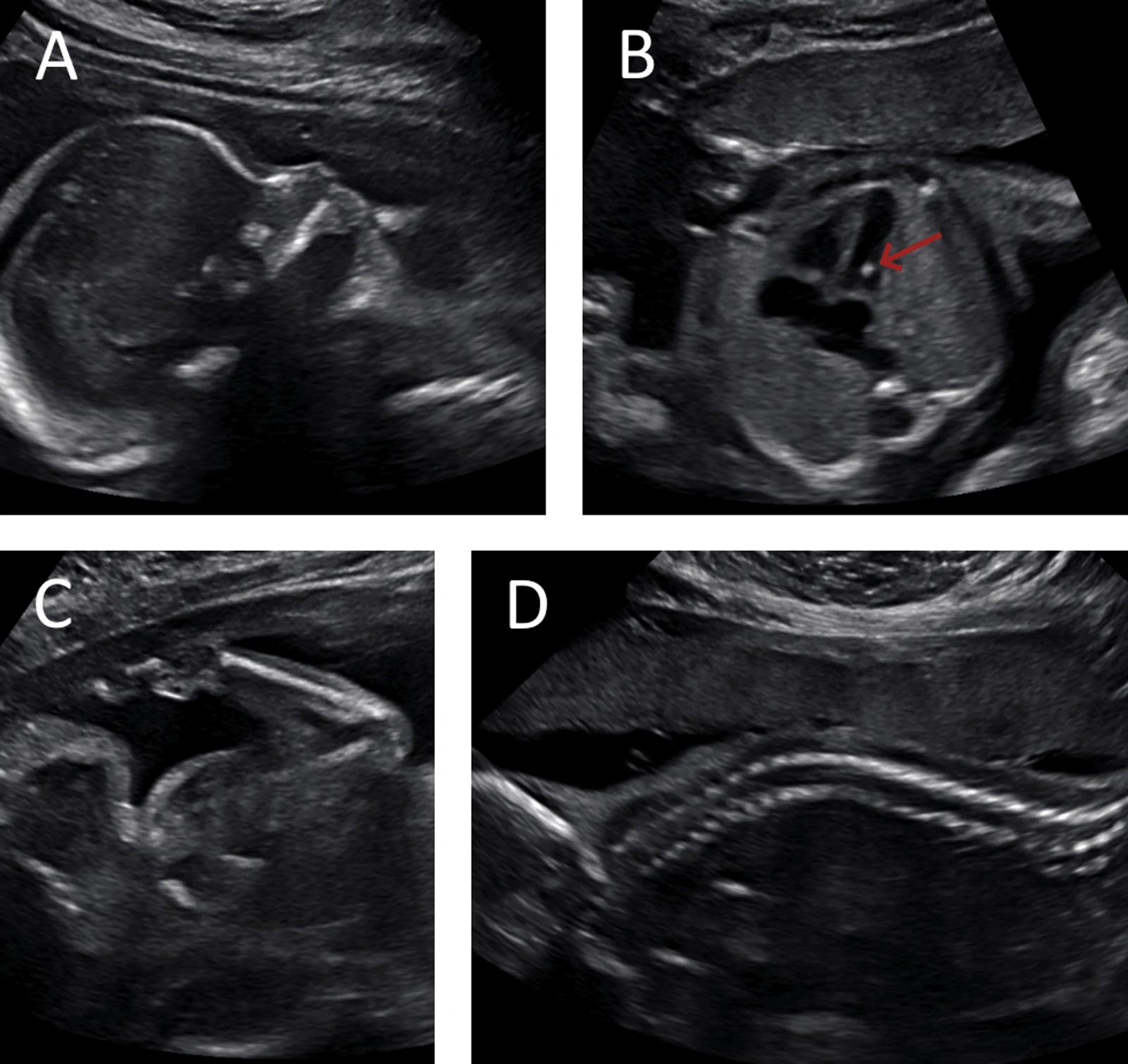
Representative images from a fetal anatomy ultrasound at an estimated 24 weeks of gestation A. Sagittal view of the head and face; B. Four-chamber view of the heart showing an echogenic focus (red arrow) but otherwise normal anatomy; C. Sagittal view of the upper body evaluating normal upper extremities, and D. Sagittal view of the spine. The ultrasound was read as normal, and the echogenic focus was no longer present in the post-natal echocardiogram.

In the third trimester, they presented with neurovisceral symptoms at 35 weeks and 1 day, concerning for an acute porphyria attack and/or hypertensive disorder of pregnancy. They were admitted to the hospital and treated with intravenous hemin and opiate analgesia. The patient’s labs are presented in Table 1. On hospital day six, they were diagnosed with preeclampsia with severe features after exhibiting two severe-range blood pressures four hours apart while on bed rest. They received one dose of labetalol as well as a magnesium sulfate bolus and subsequent continuous infusion of magnesium sulfate. Vacuum-assisted vaginal delivery was performed in the setting of recurrent variable and late decelerations. Delivery was complicated by a postpartum hemorrhage, which was quickly controlled.

**Table 1:** Patient's labs obtained upon admission

Lab	Value	Units	Reference Range
White Blood Cell Count	8.4	× 109/L	4.5 - 10.0
Red Blood Cell Count	3.24	× 1012/L	3.90 - 5.10
Hemoglobin	9.8	g/dL	12.0 - 14.6
Hematocrit	28.6	%	36.0 - 44.0
Mean Corpuscular Volume	88.3	fL	82.0 - 97.0
Mean Corpuscular Hemoglobin	30.2	pg	27.0 - 33.0
Mean Corpuscular Hemoglobin Concentration	34.2	g/dL	32.0 - 35.0
Red Cell Distribution Width	13	%	12.0 - 15.0
Platelet Count	140	× 109/L	160 - 360
Mean Platelet Volume	9	fL	7.0 - 11.0
Neutrophil Absolute	5.7	× 109/L	1.8 - 8.0
Lymphocyte Absolute	2.2	× 109/L	1.2 - 3.3
Monocyte Absolute	0.4	× 109/L	0.3 - 1.0
Eosinophil Absolute	0.1	× 109/L	0.0 - 0.4
Basophil Absolute	0	× 109/L	0.0 - 0.1
Sodium	137	mmol/L	135 - 145
Potassium	3.6	mmol/L	3.5 - 5.1
Chloride	106	mmol/L	100 - 110
Bicarbonate	22	mmol/L	20 - 30
Blood Urea Nitrogen	8	mg/dL	7 - 18
Creatinine	0.67	mg/dL	0.50 - 1.00
Glucose	95	mg/dL	65 - 99
Calcium	8.3	mg/dL	8.5 - 10.3
Magnesium	1.9	mg/dL	1.6 - 2.6
Antiglobulin Screen	Negative	-	Negative
Urine Color	Straw to yellow	-	
Urine Clarity	Cloudy	-	Clear
Urine Specific Gravity	1.023	-	1.005 - 1.030
Urine pH	6.5	-	5.0 - 8.0
Urine Protein	Trace	mg/dL	Negative
Urine Glucose	Negative	mg/dL	Negative
Urine Ketones	Negative	mg/dL	Negative
Urine Bilirubin	Negative	-	Negative
Urine Blood	Negative	-	Negative
Urine Urobilinogen	< 2.0	mg/dL	< 2.0
Urine Leukocytes	Negative	-	Negative
Urine Nitrites	Negative	-	Negative
Urine Red Blood Cells	3	/ high-power field	0 - 3
Urine White Blood Cells	4-10	/ high-power field	0 – 3
Urine Bacteria	Many	/ high-power field	None
Urine Mucous Cells	None-few	/ low-power field	None-few
Urine Squamous Epithelial Cells	> 30	/ low-power field	0 - 3
24-Hour Urine Porphobilinogen	0.095	mg/24hr	<0.34

The patient was discharged in stable condition. Twenty-four-hour urine porphobilinogen results had been sent out upon admission and ultimately returned normal, essentially excluding a porphyria attack as the admitting diagnosis. The infant required a brief stay in the neonatal ICU for respiratory depression in the setting of intrapartum magnesium exposure and prematurity. The neonate showed no congenital abnormalities or likely effects from prenatal givosiran treatment. Echocardiography revealed normal cardiac anatomy; an echogenic focus was not present. At the time of this writing, the child is three years old, remains healthy, and is meeting all developmental milestones. The infant did not inherit the *CPOX* mutation and therefore does not carry a diagnosis of hereditary coproporphyria.

## Discussion

While current guidelines consider givosiran to be front-line therapy [4,7], its risks in pregnancy are not described. We present our patient who chose to continue givosiran therapy after a thorough discussion of the risks and benefits, and frank discussion of what was and continues to be unknown. The patient remained free of neurovisceral attacks but developed preeclampsia in the third trimester. A healthy infant was delivered. To our knowledge, there have not been other reports of givosiran use in pregnancy, successful or otherwise.

Approximately 5 in 100,000 individuals will be diagnosed with hereditary porphyria [8]. Acute hepatic porphyrias (AHPs), of which HCP is one type, are hereditary disorders of heme biosynthesis that lead to a buildup of porphyrin precursors in the bloodstream [5]. In AHPs, defects in the heme biosynthesis pathway cause neurovisceral attacks mediated by a buildup of neurotoxic porphyrin precursors, including δ-aminolevulinic acid (ALA) and PBG [5].

During a neurovisceral attack of hereditary coproporphyria, a patient may experience intense abdominal pain, peripheral neuropathies, psychosis, and/or respiratory paralysis that may be fatal [8]. Individuals who experience frequent attacks, over four attacks per year on average, are at risk of developing chronic neurologic effects [3]. Drug exposure and calorie restriction are often triggers for neurovisceral attacks [9]. Management of an acute attack typically includes discontinuation of possible drug triggers [4]. For individuals with frequent acute porphyric attacks, liver transplantation may be considered if the disease is refractory to repeated hemin courses [1,7].

With regard to hormone exposure, progesterone and its metabolites, as well as synthetic progestins such as those found in various hormonal methods of contraception, are known triggers, and the increased progesterone levels mediated by the physiological changes conferred by pregnancy may have contributed to our patient’s pregnancy complications [10]. Patients with porphyria are at risk for both increased frequency of acute attacks as well as preterm delivery [11]. Pregnant people with familial porphyria cutanea tarda (PCT) have twice the risk of preeclampsia as pregnant people without the disease [12]. Furthermore, primiparas with active acute porphyria as well as those with the heritable form of PCT exhibit increased risk of perinatal death [12]. In a systematic review, approximately 71% of patients with porphyria experienced at least one acute porphyric attack during pregnancy. In this population, acute porphyric attacks were characterized by neurologic symptoms, abdominal pain, and psychiatric disturbances [11]. One study demonstrated that nearly one-third of pregnant patients with porphyria experienced preterm births. Pregnant patients with a porphyria diagnosis were also more likely to require cesarean delivery compared to the general population [11]. A Swedish study including 214 pregnant people with known AHP revealed an elevated risk for hypertensive disorders of pregnancy, gestational diabetes, and small-for-gestational age when compared to the control cohort [13]. There was no elevated risk in perinatal mortality or fetal malformations when controlling for porphyria disease status [11]. Prior to givosiran development, pregnant people were treated with hemin infusions, either prophylactically throughout gestation or only as needed during acute porphyric attacks [1].

Givosiran is processed by endonuclease Dicer to form dsRNA, then incorporated into an RNA-induced silencing complex [10]. The outcome of givosiran incorporation is the destruction of ALAS1 mRNA and therefore decreased rate of translation of ALAS1 [5]. Because ALAS catalyzes the first step in heme biosynthesis to form neurotoxic ALA and PBG, givosiran works to decrease the frequency and severity of neurovisceral attacks [5]. In the randomized phase III ENVISION trial (NCT03338816), patients receiving givosiran exhibited a 74% decrease in the frequency of neurovisceral attacks compared to placebo [14]. In the long-term crossover phase, placebo patients switching to givosiran saw a decrease in the median annualized attack rate, dropping from 10.65 to 0.87 [15].

The United States Food and Drug Administration (FDA) does not currently have an evaluation of drug-associated risk for adverse maternal-fetal outcomes, including miscarriage, in pregnant patients treated with givosiran [6]. In rabbit studies, givosiran has been shown to have a fetotoxic dosage of 1.5 mg/kg/day, which is five times the maximum human dosage [6]. The current guidance remains for patients and medical providers to exercise shared decision-making before prescribing givosiran during a pregnancy. In the above case, our patient with hereditary coproporphyria elected to continue with givosiran treatment during pregnancy. Their pregnancy remained uncomplicated prior to the third trimester, and there were no noted fetal effects of givosiran treatment. While givosiran is highly effective in preventing acute neurovisceral attacks, its safety profile in pregnancy remains undefined. This case highlights the role of shared decision-making in managing complex obstetric cases involving high-risk conditions and novel therapies.

## Conclusions

As givosiran use in pregnancy in patients with AHPs has not been well-studied, discussions about sustaining givosiran during pregnancy remain challenging for both patients and providers. This case underscores the importance of shared decision-making in complex medical scenarios, particularly when managing rare conditions with limited evidence. The patient’s decision to continue givosiran therapy during pregnancy, despite the lack of extensive safety data, was based on a clear discussion of the risks and benefits, eliciting the patient's values and facilitating informed choices. Such collaborative approaches empower patients and enhance conversations on managing serious medical conditions during pregnancy.
